# *SAR1a* promoter polymorphisms are not associated with fetal hemoglobin in patients with sickle cell disease from Cameroon

**DOI:** 10.1186/s13104-017-2502-3

**Published:** 2017-05-12

**Authors:** Gift Dineo Pule, Valentina Josiane Ngo Bitoungui, Bernard Chetcha Chemegni, Andre Pascal Kengne, Ambroise Wonkam

**Affiliations:** 1 Division of Human Genetics, Department of Pathology, Faculty of Health Sciences, University of Cape Town, Anzio Road, Observatory, Cape Town, 7925 Republic of South Africa; 2 Faculty of Medicine and Biomedical Sciences, University of Yaoundé, Yaounde, Cameroon; 3 Non-Communicable Diseases Research Unit, South African Medical Research Council, Cape Town, South Africa

**Keywords:** *SAR1a* promoter, Fetal hemoglobin, Sickle cell disease, Cameroon

## Abstract

**Background:**

Reactivation of adult hemoglobin (HbF) is currently a dominant therapeutic approach to sickle cell disease (SCD). In this study, we have investigated among SCD patients from Cameroon, the association of HbF level and variants in the HU-inducible small guanosine triphosphate-binding protein, secretion-associated and RAS-related (*SAR1a*) protein, previously shown to be associated with HbF after HU treatment in African American SCD patients.

**Results:**

Only patients >5 years old were included; hemoglobin electrophoresis and a full blood count were conducted upon arrival at the hospital. RFLP-PCR was used to describe the *HBB* gene haplotypes and Gap PCR to investigate the 3.7 kb α-globin gene deletion. The iPLEX Gold Sequenom Mass Genotyping Array and cycle sequencing were used for the genotyping of four selected SNPs in *SAR1a* (rs2310991; rs4282891; rs76901216 and rs76901220). Genetic analysis was performed using an additive genetic model, under a generalized linear regression framework. 484 patients were studied. No associations were observed between any of the promoter variants and baseline HbF, clinical events or other hematological indices.

**Conclusion:**

The results of this study could be explained by possible population-specificity of some tagging genomic variants associated with HbF production and illustrated the complexity of replicating HbF-promoting variants association results across African populations.

## Background

Reactivation of adult fetal hemoglobin (HbF) is currently the dominant approach for the treatment of sickle cell disease (SCD). The expression of adult HbF is a quantitative trait that is subject to several predisposing loci affecting the persistence of HbF in adulthood, particularly three principal loci; *BCL11A*, *HBS1L*-*MYB* intergenic variants and the five sequence polymorphisms along the β-globin gene cluster that confer the SCD haplotype [1–3]. Taken in sum, these loci have been associated with the disease-ameliorating HbF and account for 10–20% of the variance [4–7]. Furthermore, the *BCL11A* erythroid-specific enhancer variants have been shown to account for significant variance in HbF in African American [8, 9], Tanzanian [10] and Cameroonian SCD patient cohorts [11].

HbF response to hydroxyurea (HU), has been shown to be subject to a myriad of genetic variations (SNPs, signalling pathways and pharmacogenomics interactions) and environmental factors (socio-economic factors, quality of care, exposure to malaria and infections) [12]. Furthermore, some of these variants have been associated with favourable pharmacologic response to HU treatment like the small guanosine triphosphate (GTP)-binding protein, secretion-associated and RAS-related (SAR) protein [13]. SAR proteins have been shown to be critical to γ-globin expression [14] via the Giα/JNK/Jun pathway [15] in response to HU. Four variants (rs2310991; rs4282891; rs76901216 and rs76901220) in the *SAR1a* promoter were associated with significant increases in HbF levels in African American SCD patients on HU for 2 years [13]. Much like variants in *BCL11A*, *MYB* and *KLF*-*1* have been shown to be associated to Hb F levels in both HU-exposed and HU-naïve conditions [6, 12], in the present study, we have investigated the relationship between the four *SAR1a* promoter variants and baseline HbF in SCD patients from Cameroon, without any HU treatment.

### Research hypothesis

In this study, we hypothesize that selected variants in *SAR1a* promoter associated with significant increases in HbF levels in African American SCD patients on HU, are also associated with baseline HbF in SCD patients from Cameroon, without any HU treatment.

## Methods

### Study population and HbF measurement

Patients’ recruitment occurred at the Yaoundé Central Hospital and Laquintinie Hospital in Douala, Cameroon. Only clinically stable patients, 5 years and older, with no history of blood transfusion, HU treatment, or hospitalisation in the preceding 6 weeks were included and clinical events were prospectively collected (Table 1). Whole blood counts of patients and Hb electrophoresis were conducted on arrival at the hospital, initially using the alkali denaturation test (ADT) in 55.5% (*n* = *266*) of the cohort, and when it became available, high performance liquid chromatography (HPLC). In a previous study of this cohort, the SCD patient cohort was disaggregated based on the technique used for HbF assessment (HPLC vs ADT) and found similar but independently examined associations between HbF levels and *BCL11A* and *HBS1L*-*MYB* intergenic variants [5].

**Table 1:** Cohort description

Variables		Mean ± SD	Value range	Number of observations
Age (years)		17.9 ± 10.6	5–57	484
Haematological indices	RBC (10^12^/l)	2.8 ± 0.7	1.2–5.5	484
	Hb (g/dl)	7.8 ± 1.6	4.0–13.5	484
	MCV(fl)	84.6 ± 10.0	61–112	484
	MCHC (g/dl)	34.2 ± 3.8	22.3–54.0	484
	WBC (10^9^/l)	14.7 ± 6.4	3.5–48.8	484
	Lymphocytes (10^9^/l)	6.1 ± 3.3	0.87–20.4	484
	Monocytes (10^9^/l)	1.7 ± 1.4	0.1–10.9	484
	Platelets (10^9^/l)	371.5 ± 131.6	108–827	484
	HbA2 (%)	3.6 ± 2.1	0.1–18.2	484
	HbF (%)	10.1 ± 8.5	0–37.4	484
Clinical events	Vaso-occlusive crisis (No./year)	2.8 ± 3.3	0–40	484
	Consultations (No./year)	3.0 ± 3.9	0–24	484
	Hospitalisation (No./year)	1.4 ± 2.5	0–30	484
	Overt stroke	4.5%		22/484
3.7del α-globin gene genotypes	αα/αα	62.9%		304/484^a^
	αα/α3.7	28.5%		138/484^a^
	α3.7/α3.7	8.6%		42/484^a^
β-globin gene haplotypes	Benin/Benin	50.2%		243/484^a^
	Benin/Cameroon	21.7%		105/484^a^
	Benin/Atypical	4.1%		20/484^a^
	Cameroon/Cameroon	4.1%		20/484^a^

*RBC* red blood cells, *Hb* hemoglobin, *MCV* mean corpuscular volume, *MCHC* mean corpuscular haemoglobin concentration, *WBC* white blood cells, *HbA2* hemoglobin α2, *HbF* hemoglobin F

^a^Number of individuals and not alleles

### Genotyping

#### HbS mutation and *HBB* haplotypes

DNA was extracted from peripheral blood following the manufacturer’s instructions (Puregene Blood Kit, Qiagen^®^, USA). Molecular analysis to determine the presence of the sickle mutation was carried out on 200 ng DNA by PCR to amplify a 770 bp segment of the β-globin gene, followed by *Dde*I restriction analysis of the PCR product [16].

Using published primers and methods, five restriction fragment length polymorphism (RFLP) sites in the β-globin gene cluster were amplified [17] to analyse the *Xmn*I (5′Gγ), *Hind*III (Gγ), *Hind*III (Aγ), *Hinc*II (3˙’Ψβ) and *Hinf*I (5′β) for the *HBB* haplotype background [18].

#### Detection of 3.7 kb α-globin gene deletions

Using the expand-long template PCR (Roche^®^, UK), the 3.7 kb α-globin gene deletion was successfully screened following the instructions reported [19] with some modifications previously published [20].

#### SNPs

A total of 484 samples were analysed initially using SNaPshot sequencing and capillary electrophoresis (*n* = *234*), then the rest analysed using the iPLEX Gold Sequenom Mass Genotyping Array (Inqaba Biotec, South Africa). The results were validated by direct cycle sequencing of a subset (*n* = *48;* 10%) of the samples using previously reported methods [5].

### Data analysis

Descriptive statistics were obtained for all quantitative data using SPSS (IBM, USA version 21.0). A Chi squared test, with 1 degree of freedom, was used to perform the Hardy–Weinberg Equilibrium (HWE) test on the SNPs genotype with three SNPs (rs4282891; rs2310991 and rs76901216) out of HWE (p < 0.05). Using an additive genetic model, under a generalized linear regression framework, we investigated the relationship between the SNPs and HbF levels, using the R statistical package version 3.0.3 (The R Foundation for statistical computing, Vienna, Austria). Significance was set at 5%.

## Results

### Patient cohort

Table 1 summarises the main characteristics of the cohort. All patients had confirmed SCA diagnosis (HbSS) among whom 50.2% (*n* = *243*) were female and the mean age of the cohort was 17.9 years (±10.6). After genotyping β-globin gene haplotypes for 968 chromosomes, the most prevalent were Benin (72.5%, *n* = *702*) and Cameroon (19.2%, *n* = *186*). Haplotypes given in combinations, the Benin/Benin haplotype was 50.2% (*n* = *243*) and the Benin/Cameroon haplotype 21.7% (*n* = *105*) of the patient cohort (Table 1). The frequency of the 3.7 kb α-globin gene deletion (α3.7) was 22.5% (*n* = *222*) of 968 chromosomes, where 28.5 and 8.6% of patients had co-inherited a single (αα/α3.7) and double (α3.7/α3.7) deletions, respectively (Table 1). The average number of annual reported vaso-occlusive crises was 2.8 (±3.3) with a similar yearly rate of hospital consultation (3.0 ± 3.9) and mean 1.4 (±2.5) hospitalization per year. Overt stroke was reported in 4.5% (*n* = *22*) of the patients.

### No association between SNPs and HbF

The minor allele frequency (MAF) of the *SAR1a* promoter SNPs is shown in Table 2. There was no association between the four selected promoter SNPs and HbF levels in the patient cohort neither was there an association with clinical events or hematological indices. The influence of the electrophoretic technique, α-globin genotypes and β-globin haplotypes on the relationship between the SNPs and HbF levels was tested and no significant effect was observed.

**Table 2:** Association between *SAR1a* promoter polymorphisms and baseline HbF in Cameroon SCD patients

SNP	SNP position	Chromosome loci	Alleles^a^	MAF	Effect size^b^ (SE)	p value
rs2310991	−1377	10:70171890	A/C	0.334	0.03 (−0.08 to 0.13)	0.645
rs4282891	Intron 1 +100	10:70170313	A/C	0.336	0.25 (−1.61 to 2.12)	0.791
rs76901216	−809	71601084	C/G	0.295	−0.05 (−0.14 to 0.05)	0.335
rs76901220	−385	10: 71600660	G/C	0.204	−0.07 (−0.20 to 0.05)	0.265

*MAF* minor allele frequency

^a^Alleles: major/minor

^b^Effect size and range

## Discussion

Four variants (rs2310991; rs4282891; rs76901220 and rs76901216) have previously been associated with higher percent HbF and significant change in HbF levels after HU treatment for 2 years in African American SCA patients enrolled in the National Institutes of Health (NIH)’s Sickle Cell Pulmonary Hypertension Screening Study (ClinicalTrials.org; NCT00011648). It has been reported that *SAR1* is induced by HU towards the production of HbF in erythroid cells [14, 15] via activation of the Giα/JNK/Jun pathway. This and other critical signalling pathways in HU-induced HbF as well as genomic variants associated with HbS mutation and the disease course have been reviewed [12]. Given the responsiveness of *SAR1* to HU treatment and prior associations with HU-induced HbF, four promoter polymorphisms were investigated in a cohort of SCA patients not treated with HU to determine possible associations between the SNPs and baseline HbF. If these variants result in differential expression of *SAR1* and thus HbF, this loci could become a candidate for therapeutic manipulation for ameliorating SCA and other hemoglobinopathies. However, none of the selected SNPs were associated with HbF, other hematological indices or clinical events. Furthermore, the difference between the minor allele frequencies (MAF) in African American SCA patients [13] and results from the present study may be attributed to the well reported high diversity among African population [21, 22] and more specifically to admixture among African Americans, with African ancestry that could vary from 1 to 99% [23, 24]. Replication of association studies across different populations, particularly of varied genetic backgrounds and environmental settings, is imperative to understanding the complex processes of HbF production and genetic polymorphisms predisposing to persistence of adult HbF, more so in sub-Saharan Africa where the burden of disease is highest.

*SAR1a* is a known regulator of HbF expression under HU therapy [13, 14], previously demonstrated in bone marrow CD34+ and K562 cells [15]. This erythroid binding protein primarily acts through the p-JNK/Jun and GATA-2 pathways alongside a network of various other erythroid regulators for HbF activation such as MYB, BCL11A and MAPKs [12]. Much like variants in *BCL11A*, *MYB* and *KLF*-*1* have been shown to be associated to Hb F levels in both HU-exposed and HU-naïve conditions [6, 12, 25, 26], we investigated whether variants at *SAR1a* could, be equally associated with Hb F level in HU-naïve patients. This is a possible limitation of this exploratory study, but equally strength for this novel investigation with results that have implications for future researches on therapy of SCD. Indeed, showing that variants at *SAR1a* are not associated in HbF regulation at steady state in SCD patients could be consider interesting; the findings provide further evidence of the complex nature of Hb F regulation, highlighting that steady-state genetic regulators of γ-globin expression are not necessarily best suited targets for therapeutic interventions, therefore suggesting widening of the search for drug-responsive targets for HbF activation.

## Conclusion

The present study did not find any association between selected SNPs in the HU-inducible *SAR1a* promoter polymorphisms, and HbF among SCD patients from Cameroon. The findings provide further evidence of the complex nature of HbF regulation, highlighting that steady-state regulators of γ-globin expression are not necessarily best suited targets for therapeutic interventions, therefore suggesting widening of the search for drug-responsive targets for HbF activation. The results of this study also emphasize the genetic heterogeneity of populations of African ancestry affected by SCD, with regard to HbF-promoting loci, and the need to perform replication studies of key association findings in several SCD populations to fully capture their significance.

## Abbreviations

HbF: hemoglobin F
SCD: sickle cell disease
HU: hydroxyurea
GTP: guanosine triphosphate
SAR: secretion-associated and RAS-related
RFLP-PCR: restriction fragment-length polymorphism polymerase chain reaction
HBB: hemoglobin beta
SNP: single nucleotide polymorphism
BCL11A: B-cell lymphoma/leukemia 11A
HBS1L: hemoglobin S-1 like translational protein
MYB: myeloblastosis protein family
Giα/JNK/Jun: mitogen-activated protein kinase pathway
ADT: alkaline denaturation test
HPLC: high performance liquid chromatography
HWE: Hardy–Weinberg equilibrium
MAF: minor allele frequency
NIH: National Institutes of Health
